# Portal Vein Recanalization–Transjugular Intrahepatic Portosystemic Shunt (PVR-TIPS) with superior mesenteric vein access and balloon-assisted shunt placement

**DOI:** 10.1186/s42155-023-00379-6

**Published:** 2023-06-08

**Authors:** Cornelia L. A. Dewald, Frank K. Wacker, Benjamin Maasoumy, Jan B. Hinrichs

**Affiliations:** 1 Institute for Diagnostic and Interventional Radiology, Hannover Medical School, Hannover, 30625 Germany; 2 Department of Gastroenterology, Hepatology, Infectious Diseases and Endocrinology, Hannover Medical School, Hannover, 30625 Germany

**Keywords:** Balloon-assisted PVR-TIPS, Portal vein occlusion, Portal Vein Recanalization–Transjugular Intrahepatic Portosystemic Shunt (PVR-TIPS), Transmesenteric access

## Abstract

**Background:**

To report the technique and outcome of ultrasound-guided percutaneous access to the superior mesenteric vein (SMV) for balloon-assisted portal vein recanalization–transjugular intrahepatic portosystemic shunt (PVR-TIPS) in a patient with chronic portal venous and splenic vein occlusion.

**Case presentation:**

A 51-year-old, non-cirrhotic patient with severe portal hypertension was admitted for PVR-TIPS. Neither splenic nor hepatic access was feasible due to chronic portal and splenic vein occlusion. Percutaneous ultrasound-guided direct puncture of the SMV was performed to obtain access for balloon-assisted PVR-TIPS. The transmesenteric approach in combination with a balloon puncture technique for PVR-TIPS was successful, and no immediate complications were observed post-procedure. The subsequent follow-up exams showed patent TIPS and SMV without signs of intraabdominal hemorrhage.

**Conclusion:**

Percutaneous ultrasound-guided superior mesenteric vein access for balloon-assisted PVR-TIPS is a feasible option in cases where hepatic or splenic access is not.

## Background

Portal vein thrombosis (PVT) can result in chronic occlusion of the portal vein (PV) [1], which might cause portal hypertension with a consecutive increase in morbidity [2]. Especially in cases with sequalae secondary to portal hypertension, portal vein recanalization (PVR) and creation of a transjugular intrahepatic portosystemic shunt (TIPS) are promising options to treat both chronic occlusion of the PV and portal hypertension [2, 3]. PVR-TIPS re-establishes portal venous flow and decompresses portal circulation [4, 5]. To gain access to the portal venous system is the pivotal step in PVR-TIPS. In recent years, shift from trans-hepatic to trans-splenic access to the portal vein has been evident [6]. However, in some cases neither the trans-splenic nor trans-hepatic routes are feasible. A few case reports describe the mesenteric veins as a potential alternative access route for snare device-guided PVR-TIPS [4, 7]. We report on a case of balloon-assisted PVR-TIPS using percutaneous ultrasound-guided superior mesenteric vein (SMV) access.

## Case presentation

**Fig. 1:**
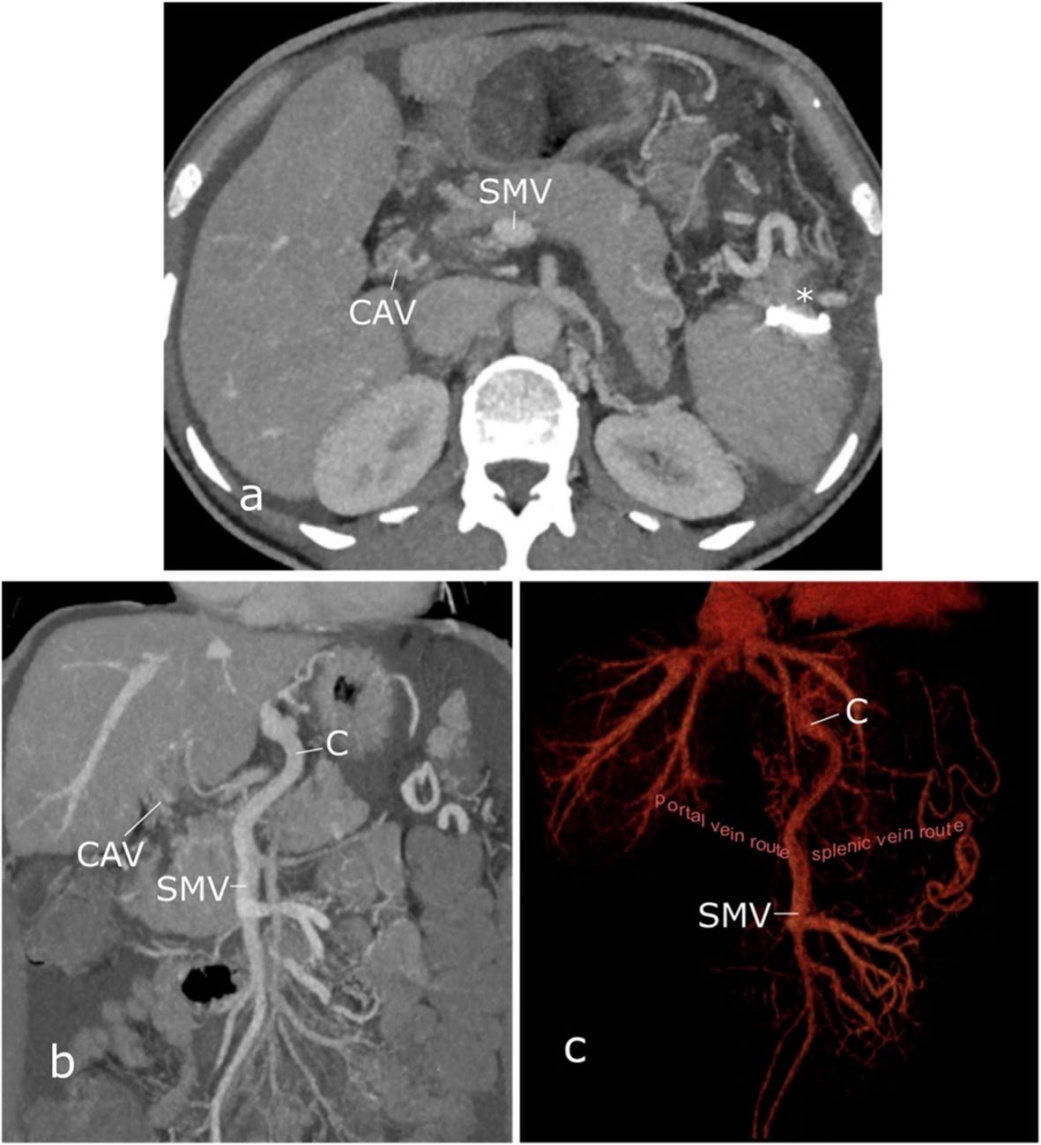
Pre-interventional computed tomography (CT) shows chronic occlusion of the portal vein and splenic vein. Cavernous transformation of the portal vein is evident (CAV). Distinct portosystemic collaterals are seen, especially periesophageally (C). The asterisk marks a coil inserted in the spleen during the previous attempt. **a** transversal view with maximum intensity projection (5 mm) (**b**) coronal view with maximum intensity projection (10 mm) (**c**) coronal view with 3D volume rendering technique reconstruction. The presumed vascular course of the occluded portal vein and splenic vein have been added manually. SMV: superior mesenteric vein

A 51-year-old female patient (BMI 20.6 kg/m^2^) with no history of hepatic cirrhosis or malignoma presented with chronic occlusion of the PV in the setting of essential thrombocythemia. Pre-interventional imaging presented an occlusion of the intrahepatic and extrahepatic PV as well as the splenic vein (SV). The PV, which could only be delineated in strands, was cavernously transformed. Dilated esophageal and gastric portosystemic collaterals, splenomegaly and ascites were evident signs of portal hypertension (Fig. 1).

Recurring esophageal bleeding had been treated with band ligation and blood transfusions. An initial percutaneous trans-splenic approach attempted in an outside hospital was aborted as the central splenic vein occlusion could not be passed.

PVR-TIPS was performed under general anesthesia. An initially attempted percutaneous trans-hepatic approach was impossible due to chronic occlusion of the central SV. In a preceding ultrasound examination, a suitable central branch of the SMV had already been identified. Given the overall slender physique of the patient, the SMV and its central branches could be visualized only few centimeters below the abdominal wall. We selected a window (I) that was free of intestinal superimposition, (II) where the vein was large enough for the insertion of a sheath and (III) that was distant enough to avoid an obstruction of the origin of the PV by the sheath. The percutaneous puncture was performed under ultrasound-guided puncture of the branch using a 4-Fr micro-puncture set (4-F Custom Procedure Kit, Merit Medical). No intestine was punctured. Subsequently, a 4-Fr sheath ( Avanti®+ , Cordis) was introduced.

**Fig. 2:**
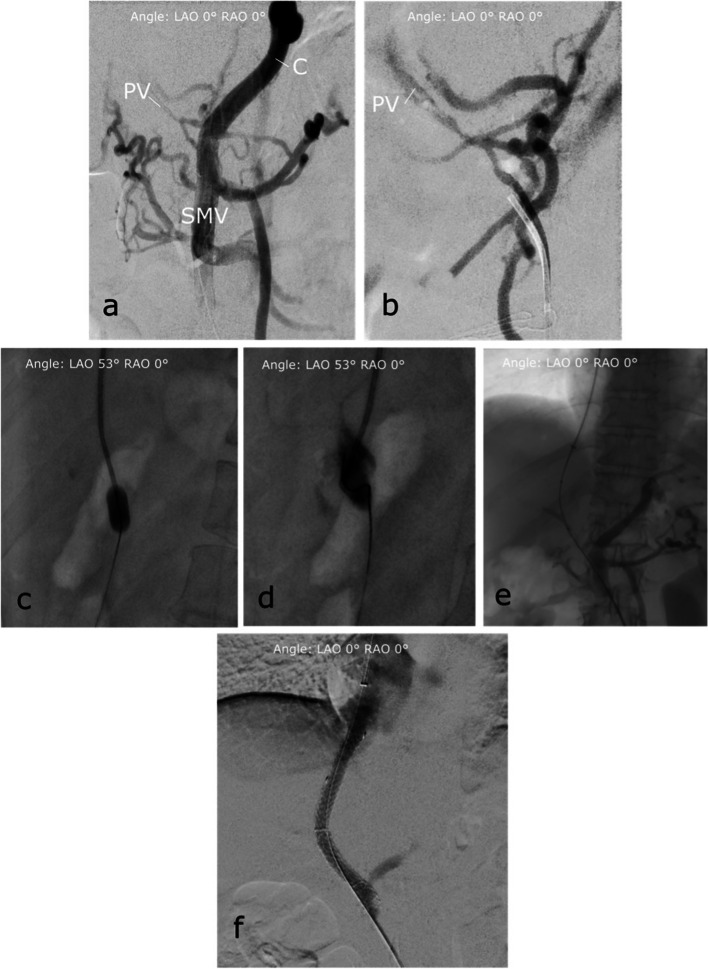
DSA and fluoroscopic images of the Portal Vein Recanalization–Transjugular Intrahepatic Portosystemic Shunt (PVR-TIPS) with percutaneous ultrasound-guided superior mesenteric vein (SMV) access and balloon-assisted shunt placement. **a** Illustrates the pre-interventional findings: In the overview angiography via the sheath in the SMV, in the presence of known chronic occlusion, there is no contrast of the splenic vein. The portal vein (PV) can only be delineated in strands and is cavernously transformed (see also the magnified image in (**b**)). In addition, evidence of multiple portosystemic collaterals, especially extending paraesophageally (C). After probing of the right PV branch was successful, a balloon catheter was positioned in the main stem of the right PV to serve as target for TIPS needle puncture (**c**). Puncture of the balloon cover was eventually successful (**d**) and Archimedean (through and through) access was achieved (**e**). After deployment of the TIPS, a rapid contrast media outflow via the TIPS and a caliber reduction of the portosystemic collaterals is evident (**f**)

After initial imaging of the occluded PV/SV and the extensive cavernous transformation (Fig. 2), passage of the PV occlusion and probing of the right portal vein branch was successful. Using a recently described balloon puncture technique, a balloon catheter (7 mmx40 mm, 135 cm, Sterling, Boston Scientific) was positioned in the main stem of the right PV to serve as target for the TIPS needle puncture (Fig. 2) [2, 8]. Afterwards, a TIPS needle (GORE TIPS Set, W.L.Gore & Associates) was advanced through a standard transjugular access into the right hepatic vein. Puncture of the balloon cover within the PV branch was hampered due to repeated slippage of the TIPS needle on the balloon surface but was finally successful (Fig. 2). A 0.014-inch wire (V-14, Control Wire, Boston Scientific) was advanced through the needle and locked within the balloon cover by deflation of the balloon.

In order to establish through and through access (Archimedean access), the balloon catheter was pulled into the direction of the SMV sheath, but the wire could not be retrieved due to severe kinking within the balloon and eventually broke. The distal end of the wire was secured through the sheath. The 4-Fr sheath in the SMV was replaced by a 6-Fr sheath and a 6-Fr multi snare catheter (EN Snare® Endovascular Snare, Merit Medical) was inserted to snare the wire. Nonetheless, the proximal end of the wire could not be retrieved, and the wire had to be removed via the jugular access. Now with the 6F sheath in place the maneuver was repeated using another balloon catheter (10 mmx40 mm, 80 cm, Mustang, Boston Scientific) positioned in the right hepatic vein and inflated as fluoroscopic target. Direct TIPS needle-guided puncture of the balloon was performed without complications, and a 0.035 wire (Splash, Merit Medical) was used to successfully achieve through and through access.

Following this, sequential widening of the TIPS tract and portal vein was performed using 6 mm and 8 mm balloons (Mustang, 6/8mmx40mm, 75 cm, Boston Scientific). Subsequent implantation of a 7/2 VIATORR endoprosthesis, which needed to be extended to the caval vein by a second 7/2 VIATORR endoprosthesis as a stent-in-stent (GORE Viatorr TIPS Endoprosthesis with controlled Expansion, W.L.Gore&Associates) was performed. The final angiography showed a swift contrast outflow via the TIPS as well as a significant caliber reduction of the collaterals (Fig. 2). The portosystemic gradient decreased from 23 to 3 mmHg. At the end of the procedure, the SMV sheath was removed, and manual compression was performed. No dedicated vascular closure was used.

**Fig. 3:**
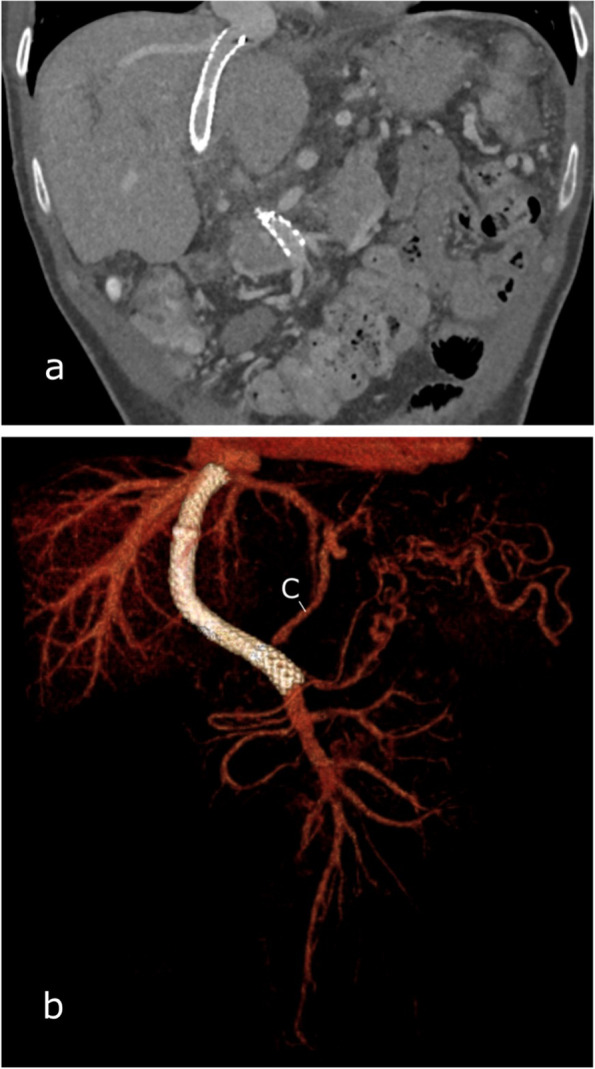
Post-interventional computed tomography (CT) on the day after TIPS insertion. The TIPS is patent, and the portosystemic collaterals have decreased in size (C). **a** Coronal view with maximum intensity projection (5 mm). **b** Coronal view with 3D volume rendering technique reconstruction

Anticoagulation with low-molecular-weight heparin was started immediately after the PVR-TIPS. For the pre- and post-interventional coagulation status see Table 1. The following day, CT showed a patent TIPS and no signs of intestinal damage or intraabdominal hemorrhage (Fig. 3). Consecutive ultrasound exams on the subsequent days were also unremarkable. The patient spent one night in intensive care and was discharged four days after the procedure. At the first follow-up one month after the procedure, the TIPS was patent; moreover, no bleeding episode or signs of hepatic encephalopathy were observed.

**Table 1:** Pre- and post-interventional coagulation status

	**Before PVR-TIPS**	**Day 1 after PVR-TIPS**
Hemoglobin (g/dl)	8.6	8.4
Platelet count (1000/µl)	238	225
Quick (%)	81.2	67.3
PTT (s)	31.6	50.2

*PVR-TIPS* Portal vein recanalization–transjugular intrahepatic portosystemic shunt, *PTT* Partial thromboplastin time

## Discussion/conclusion

Treatment of PV occlusion due to PVT is challenging, and management is done on a case-by-case basis. In cirrhotic and non-cirrhotic patients predisposed to venous thrombosis, anticoagulation has been suggested [4, 9]. However, the high bleeding risk due to the development of esophageal and gastric varices in many cases of chronic PV occlusion is a significant challenge and balancing anticoagulation with the risk of bleeding can be problematic [3].

PVR-TIPS has been shown to reduce the risk of bleeding over 2 years in patients with thrombotic PV occlusion compared to alternatives such as esophageal band ligation and propranolol [3]. In regard to accessing the PV for reconstruction in PVR-TIPS, the percutaneous trans-hepatic method comes with a couple of drawbacks, e.g., that the approach is predicated on the intrahepatic PV not being fully occluded [7, 10]. The trans-splenic approach has become the technique of choice in PVR-TIPS, but similar to the trans-hepatic approach, it also has its limitations, e.g., in thrombotic SV or asplenic patients. Furthermore, the vascular anatomy of the splenic vein may influence the technical execution of PVR-TIPS, whereas the SMV is nearly vertically in line with the PV, which can have a positive effect on maneuverability within the portal system [7].

There are a few case reports exploring trans-mesenteric access via minilaparotomy [11, 12], a method that has not gained wide popularity (probably due to the high complication rate) [7, 13]. In a more recent case series, Entezari et al. reported on four patients treated with PVR-TIPS via percutaneous ultrasound-guided superior and inferior mesenteric vein access using a gooseneck snare as a target for TIPS needle puncture (gun-sight technique [14]) with encouraging results [4]. Another recent case study reports one case of percutaneous CT-guided superior mesenteric vein access (also with loop snare guided TIPS needle puncture) [7]. Hence, little data is available on this novel access route and no data is available on the combination of SMV access and balloon-assisted TIPS needle placement. With our experience limited to one case so far, we agree with the authors' opinion that in individual cases access via the SMV is technically possible and associated with a good early outcome.

During the procedure, we experienced difficulties when puncturing the balloon catheter with the TIPS needle. These difficulties are probably related to the steep puncture with a corresponding limited bending of the TIPS needle. It could also be related to the fact that our patient was liver healthy and therefore had soft liver tissue. This theory might be supported by another study, in which we demonstrated complication-free balloon-assisted TIPS needle punctures in 14 patients with cirrhotic (and thus harder) liver parenchyma [2]. Especially in challenging cases, knowledge of conservative techniques such as the gun-sight technique can be helpful.

This case study is reporting on one patient only; thus our results and assumptions must be taken with great caution. However, in contrast to the previously published case reports, we inserted a sheath with a larger lumen (6-Fr) in the SMV (Entezari et al.: 4-Fr; Ghosh et al.: 5-Fr) [4, 7]. In our patient, no complications occurred, even though no measures were taken to perform vascular closure and though anticoagulation with low-molecular-weight heparin for prophylaxis of TIPS thrombosis was started immediately after the intervention. Widely used vascular closing devices, though out of instructions for this use, could be discussed but should be considered very carefully as potentially interposed intestine could be severely affected.

To conclude, percutaneous trans-mesenteric access is a feasible alternative to perform PVR-TIPS with preliminary favorable clinical success and safety. Furthermore, the combination of SMV access and balloon assisted shunt placement is a new addition to the portfolio in this often technically demanding intervention. Nonetheless, further studies should evaluate long-term safety and clinical outcome of this novel procedure.

## Data Availability

Data and materials are available at request. Requests should be addressed to the corresponding author C.L.A.D.

## Abbreviations

PVT: Portal vein thrombosis
PV: Portal vein
PVR: Portal vein recanalization
TIPS: Transjugular intrahepatic portosystemic shunt
SMV: Superior mesenteric vein
SV: Splenic vein
